# Figure-based speech perception test: applicability in children with Down syndrome

**DOI:** 10.1590/2317-1782/20212020204

**Published:** 2022-01-07

**Authors:** Beatriz Nascimento Gonçalves, Isabela Raymundini Lorenssete, Nicolle Oliveira Tomé, Ana Lúcia Rios Mota, Cristiane Fregonesi Dutra Garcia, Ana Cláudia Mirândola Barbosa Reis

**Affiliations:** 1 Curso de Fonoaudiologia, Departamento de Fonoaudiologia, Faculdade de Medicina – FM, Universidade Federal do Rio de Janeiro – UFRJ - Rio de Janeiro (RJ), Brasil.; 2 Curso de Fonoaudiologia, Departamento de Ciências da Saúde, Faculdade de Medicina de Ribeirão Preto – FMRP, Universidade de São Paulo – USP - Ribeirão Preto (SP), Brasil.

**Keywords:** Auditory perception, Child, Hearing, Down Syndrome, Speech Therapy, Speech Perception

## Abstract

**Purpose:**

To verify the applicability of the picture-based speech perception test in children with Down syndrome.

**Methods:**

Observational, descriptive, prospective study, carried out at two speech therapy centers, approved by their Research Ethics Committees under numbers 82522217.5.0000.5440 and 79510317.8.0000.5257. A total of 41 children with Down syndrome, of both sexes, aged 2 years to 10 years and 11 months participated. They were divided into three groups: GI (2 years to 4 years and 11 months); GII (5 years to 7 years and 11 months); GIII (8 years to 10 years and 11 months). We verified their medical history and carried out meatoscopy, pure-tone threshold audiometry, speech recognition threshold test with pictures, and immittance tests. For statistical analysis, we used Fisher's Exact Test with the 5% significance level.

**Results:**

The analysis of hits and misses in relation to chronological age revealed significance in seven words: “ice”, “knife”, “cow”, “key”, “mouse”, “dog”, and “sun”. We then analyzed this study participants’ performance in the speech test with pictures and those in the study that developed and validated this test. Comparing the percentage of correct answers in the two groups, we found that the words with the most correct answers were “hand”, “house”, and “frog”.

**Conclusion:**

The test applied in this study provides a clear and objective interpretation of the results, regardless of the child's verbal production.

## INTRODUCTION

Down syndrome (DS) is a genetic disorder characterized by the presence of an extra copy of chromosome 21 or an excess of the genetic material of this chromosome^(1)^. Studies have shown that this syndrome occurs in one per 1,000 live births^(2)^. The most common clinical features are intellectual disability, muscular hypotonia, slanted eyes, increased vascularity, microcephaly, and flattened occiput. Mucus accumulation may result in upper airway infections and a consequent increase in otitis media^(3)^. Middle ear dysfunction or involvement, which is frequent in this population, may also contribute to the increase in otitis media. This factor is related to anatomical malformations, such as an abnormal auditory tube, persistent mesenchymal tissue in the tympanic cavity, stenosis of the external auditory canal, and mastoid hypoplasia^(4)^.

The first years of life are important for language development, which may be abnormal in children with recurrent otitis media, impairing school learning^(5)^. Hearing is of paramount importance for the acquisition of oral language. The prerequisites for normal language acquisition are anatomical and functional integrity of the peripheral and central auditory systems and exposure to auditory experiences. The first years of life, especially the first six months, are critical for the development of auditory skills. In hearing children, auditory development and maturation follow a standardized sequence of behaviors that evolve from birth to 2 years old, namely: detection, discrimination, localization, recognition, and auditory comprehension skills^(6,7)^.

Children with DS can have intellectual disability, which, in addition to hearing loss, can lead to difficulties in language and speech development. Children need the full auditory pathway to have access to clear information and so grasp the meaning of what they hear. Sensory deprivation prevents them from developing auditory skills in their natural course^(8)^. For this population, hearing loss throughout childhood can hinder auditory skill development and consequently lead to impaired auditory processing^(9)^.

It is challenging to evaluate auditory structures and their functioning in childhood, especially in children with DS. Various procedures and techniques, including behavioral and objective ones, are used in the investigation. Aiming to make speech assessment more playful and effective, a graphic instrument was developed to test children’s speech perception. It is quick and easy to apply and has proved to be efficient in meeting the objectives in the child population since it can be applied regardless of verbal ability^(10)^.

Speech perception tests designed for children should use familiar words and be orally administered, making stimuli presentation easier during their attention span. We must develop standardized protocols and procedures to evaluate specific aspects of Portuguese speech sound perception^(11)^. Children with DS may have deficits in language and speech development, impaired by cognitive disability and/or hearing change. We should also value easy-to-use playful assessment instruments that favor quantitative/qualitative responses. Thus, this study aimed to verify the applicability of the picture-based speech perception test in children with DS.

## METHODS

The research was approved by the originating institutions’ Research Ethics Committees under CAAE numbers 82522217.5.0000.5440 and 79510317.8.0000.5257.

This is an observational, descriptive, prospective study. All participants and their parents/guardians signed the Informed Consent Form.

We assessed children diagnosed with DS, of both sexes, aged 2 to 10 years. The children were divided into three groups based on their age. Group GI comprised children aged 2 years to 4 years and 11 months, GII comprised children aged 5 years to 7 years and 11 months, and GIII comprised children aged 8 years to 10 years and 11 months.

Children who did not meet the inclusion criteria and whose parents/guardians did not agree to participate or refused to complete the assessments were excluded.

The study consisted of several steps. Firstly, we surveyed the parents/guardians regarding the children’s audiological, medical, and otologic history. Then, we inspected the external acoustic meatus and tympanic membrane with an otoscope to confirm whether an audiological assessment was feasible.

All the participants also underwent pure-tone threshold audiometry in an acoustically treated booth at 500, 1000, 2000, and 4000 Hz. We presented the warble stimulus with an audiometer, descending 10 dB and ascending 5 dB at a time to find the hearing threshold. The audiometric threshold was considered normal based on criteria from the World Health Organization^(12)^ and not the minimum response level. The procedures were carried out in a free-field system at 0º azimuth, with the loudspeaker positioned 60 cm away from the ear pinna – in case the participants refused to use supra-aural phones.

We also conducted the Speech Recognition Threshold (SRT) to confirm the pure-tone threshold, using simple commands, such as: “Where's the head”, “Blow me a kiss”, “Say goodbye”. The initial intensity was 40 dBSL (decibel sensation level) at the three-frequency mean identified in the audiological assessment, proceeding with the descending/ascending method.

After that, we applied the Speech Recognition Percentage Index (SRPI) with pictures^(10)^, at the same intensity. It consists of one training and five testing sheets, with six pictures in each corresponding to monosyllable and disyllable words; the child is expected to point at the picture related to the word the examiner said. Only five words from each sheet were used to avoid the sixth one from being an obvious answer. For this procedure, either a second examiner or the child’s parent/guardian (properly trained) went into the booth and showed the sheets corresponding to the words. The child was previously trained with a list of five words (training sheet). The word list was applied without pauses in between phonemes, repeated only once, if necessary. Introductory phrases were used to present the pictures, for example: “Show me the _______” and “Where is the _______”. The child was kept from seeing the examiner’s mouth to avoid speechreading, as in the conventional SRPI test. After the audiological assessment, the child was taken to immittance testing to verify the functioning of the tympanic-ossicular chain and investigate the contra- and ipsilateral acoustic reflexes. For the statistical analysis, we used the R Studio software and Fisher's Exact test at the 5% significance level.

## RESULTS

The research sample consisted of 41 children with DS, aged 2 years to 10 years and 11 months. Of the total sample, 25 children were assessed at the Department of Speech Therapy at the Clementino Fraga Filho University Hospital (HUCFF) of the Federal University of Rio de Janeiro (UFRJ) and the other 16, at the Specialized and Speech Therapy Center at the Clinics Hospital of the Ribeirão Preto School of Medicine of the University of São Paulo (CEOF - HCFMRP - USP). The children were divided into three groups based on chronological age. Group GI comprised children aged 2 years to 4 years and 11 months, GII comprised children aged 5 years to 7 years and 11 months, and GIII comprised children aged 8 years to 10 years and 11 months. GI was the largest group, with 21 children (51%), followed by GIII, with 12 children (29%), and GII, with 8 children (20%). The descriptive data (percentage, mean, median, standard deviation, and minimum/maximum values) regarding the children’s age per group are shown in Table 1.

**Table 1:** Descriptive data regarding the participants’ age, per group, in years (n=41)

Groups	n	(%)	Mean (years)	SD (years)	Minimum (years)	Median (years)	Maximum (years)
GI	21	51%	3.2	0.88	2	3	4.11
GII	8	20%	6.3	0.75	5.1	6.5	7.4
GIII	12	29%	9.4	0.82	8	9.2	10.6

**Caption:** n = number of children; (%) = percentage; SD = standard deviation; GI = Group 1; GII = Group 2; GIII = Group 3

The groups were composed of children of both sexes. Most girls were in GIII (37%). Altogether, 22 boys (54%) and 19 girls (46%) participated.

We could not perform pure-tone audiometry in most of the participants (60%), as they did not understand how to proceed – perhaps because they may have cognitive and language delays. On the other hand, we performed the SRT in 73% of them (n=30), most of whom (51%) had normal thresholds. The audiological assessment data are shown in Table 2.

**Table 2:** Data from the audiological assessment performed before the picture-based speech test

Variables		n=41	(%)
Free-Field Audiometry	15 dB	16	40%
Hearing thresholds RE	Not performed	15	36%
	Up to 15 dB	5	12%
	More than 15 dB	5	12%
Hearing thresholds LE	Not performed	15	36%
	Up to 15 dB	2	5%
	More than 15 dB	8	19%
SRT RE	Not performed	11	27%
	Up to 15 dB	21	51%
	More than 15 dB	9	22%
SRT LE	Not performed	11	27%
	Up to 15 dB	21	51%
	More than 15 dB	9	22%

**Caption:** n = number of children; (%) = percentage; RE = right ear; LE = left ear; SRT = Speech Recognition Threshold

The tympanometry revealed abnormal results regarding the tympanic-ossicular system of about 65% of the sample. Types B and C curves were the most frequent ones, followed by As and Ad, in one of the ears. We found normal middle ear conditions (type A curve) in 20% of the participants, while 15% of the sample did not allow us to examine them. Many of the participants refused to put on the headphone to investigate their contra- (58%) and ipsilateral (49%) acoustic reflexes. Table 3 shows the middle ear assessment data.

**Table 3:** Data on the sample’s tympanometry and reflex research

Variables		n=41	(%)
RE Tympanometry	Type A Curve	8	20%
	Type B Curve	14	34%
	Type C Curve	9	21%
	Type As Curve	3	7%
	Type Ad Curve	1	3%
	Not performed	6	15%
LE Tympanometry	Type A Curve	9	21%
	Type B Curve	13	32%
	Type C Curve	8	20%
	Type As Curve	3	7%
	Type Ad Curve	1	3%
	Not performed	7	17%
RE Ipsilateral Reflex	Not performed	20	49%
	Present	6	15%
	Absent	15	36%
LE Ipsilateral Reflex	Not performed	20	49%
	Present	5	12%
	Absent	16	39%
RE Contralateral reflex	Not performed	24	58%
	Present	6	15%
	Absent	11	27%
LE Contralateral reflex	Not performed	24	58%
	Present	6	15%
	Absent	11	27%

**Caption:** n = number of children; (%) = percentage; RE = right ear; LE = left ear

Only 32 of the 41 participants responded to the picture-based speech perception test; thus, we reduced the total sample n for the result analysis. Table 4 describes the percentages of correct answers obtained in the picture-based speech perception test per word. The group performance analysis revealed a higher rate of correct answers in GIII (90%), followed by GII and GI.

**Table 4:** Description of the sample’s hits and errors per word in the picture-based SRPI (n=32)

Pictures		Right	Wrong
	n	28	4
Duck	%	87.5%	12.5%
	n	27	5
Ball	%	84.4%	15.6%
	n	27	5
Tennis	%	84.4%	15.6%
	n	26	6
Finger	%	81.2%	18.7%
	n	29	3
House	%	90.6%	9.4%
	n	27	5
Cat	%	84.4%	15.6%
	n	8	24
Ice	%	25%	75%
	n	14	18
Knife	%	43.8%	56.2%
	n	25	7
Cow	%	78.1%	21.9%
	n	29	3
Toad	%	90.6%	9.4%
	n	18	14
Zebra	%	56.2%	43.8%
	n	23	9
Key	%	71.8%	28.2%
	n	24	8
Motorcycle	%	75%	25%
	n	27	5
Lion	%	84.4%	15.6%
	n	21	11
Mouse	%	65.6%	34.4%
	n	17	15
Ring	%	53.1%	46.9%
	n	30	2
Eye	%	93.7%	6.3%
	n	23	9
Grape	%	71.8%	28.2%
	n	27	5
Foot	%	84.4%	15.6%
	n	23	9
Train	%	71.8%	28.2%
	n	15	17
Dog	%	46.9%	53.1%
	n	26	6
Flower	%	81.2%	18.7%
	n	22	10
Sun	%	68.7%	31.3%
	n	30	2
Hand	%	93.7%	6.3%
	n	21	1
King	%	65.6%	34.4%

**Caption:** (n) = number; (%) = percentage

The 32 participants’ performance per word in the test was not 100% correct. Table 5 shows the descriptive data regarding their performance in the SRPI with pictures, namely: number and percentage of correct and incorrect answers, frequency, and percentage values, per word. The children had a better performance in the words “eye”, “hand”, “house”, “frog”, and “duck”. On the other hand, they had greater difficulty recognizing the pictures related to “ice”, “knife”, “dog”, “ring”, and “zebra” (in decreasing order of occurrence), whose error rate was, consequently, higher.

**Table 5:** Number of correct and incorrect answers in the picture-based SRPI associated with the participants’ age in GI, GII, and GIII (n=32)

Pictures			Groups			Fisher's Exact Test
			GI	GII	GIII	
			(n=14)	(n=6)	(n=12)	
Duck	Right	n	12	5	11	
		%	85.7%	83.3%	91.7%	p=1.000
	Wrong	n	2	1	1	
		%	14.2%	16.6%	8.3%	
Ball	Right	n	10	5	12	
		%	71.4%	83.3%	100%	p=0.397
	Wrong	n	2	1	0	
		%	14.2%	16.6%	0%	
Tennis	Right	n	10	5	12	
		%	71.4%	83.3%	100%	p=0.136
	Wrong	n	4	1	0	
		%	28.5%	16.6%	0%	
Finger	Right	n	9	6	11	
		%	64.2%	100%	91.7%	p=0.149
	Wrong	n	5	0	1	
		%	35.7%	0%	8.3%	
House	Right	n	12	5	12	
		%	85.7%	83.3%	100%	p=0.390
	Wrong	n	2	1	0	
		%	14.2%	16.6%	0%	
Cat	Right	n	10	5	12	
		%	71.4%	83.3%	100%	p=0.136
	Wrong	n	4	1	0	
		%	28.5%	16.6%	0%	
Ice	Right	n	1	1	6	
		%	7.1%	16.6%	50%	p=0.042*
	Wrong	n	13	5	6	
		%	92.8%	83.3%	50%	
Knife	Right	n	2	3	9	
		%	14.2%	50%	75%	p=0.005*
	Wrong	n	12	3	3	
		%	85.7%	50%	25%	
Cow	Right	n	9	4	12	
		%	64.2%	66.7%	100%	p=0.045*
	Wrong	n	5	2	0	
		%	35.7%	33.4%	0%	
Toad	Right	n	12	5	12	
		%	85.7%	83.3%	100%	p=0.390
	Wrong	n	2	1	0	
		%	14.2%	16.6%	0%	
Zebra	Right	n	5	4	9	
		%	35.7%	66.7%	75%	p=0.146
	Wrong	n	9	2	3	
		%	64.2%	33.4%	25%	
Key	Right	n	8	3	12	
		%	57.1%	50%	100%	p=0.012*
	Wrong	n	6	3	0	
		%	42.8%	50%	0%	
Motorcycle	Right	n	9	5	10	
		%	64.2%	83.3%	83.4%	p=0.563
	Wrong	n	5	1	2	
		%	35.7%	16.6%	16.6%	
Lion	Right	n	10	5	12	
		%	71.4%	83.3%	100%	p=0.136
	Wrong	n	4	1	0	
		%	28.5%	16.6%	0%	
Mouse	Right	n	5	4	12	
		%	35.71%	66.7%	100%	p=0.001*
	Wrong	n	9	2	0	
		%	64.2%	33.4%	0%	
Ring	Right	n	5	3	9	
		%	35.7%	50%	75%	p=0.097^#^
	Wrong	n	9	3	3	
		%	64.2%	50%	25%	
Eye	Right	n	12	6	12	
		%	85.7%	100%	100%	p=1.000
	Wrong	n	2	0	0	
		%	14.2%	0%	0%	
Grape	Right	n	8	5	10	
		%	57.1%	83.3%	83.4%	p=0.349
	Wrong	n	6	1	2	
		%	42.8%	16.6%	16.6%	
Foot	Right	n	11	5	11	
		%	78.2%	83.3%	91.7%	p=0.229
	Wrong	n	3	1	0	
		%	21.4%	16.6%	0%	
Train	Right	n	8	5	10	
		%	57.1%	83.3%	83.4%	p=0.349
	Wrong	n	6	1	2	
		%	42.8%	16.6%	16.6%	
Dog	Right	n	5	1	9	
		%	35.7%	16.6%	75%	p=0.042*
	Wrong	n	9	5	3	
		%	64.2%	83.3%	25%	
Flower	Right	n	9	5	12	
		%	64.2%	83.3%	100%	p=0.061^#^
	Wrong	n	5	1	0	
		%	35.7%	16.6%	0%	
Sun	Right	n	6	5	11	
		%	42.8%	83.3%	91.7%	p=0.022*
	Wrong	n	8	1	1	
		%	57.1%	16.6%	8.3%	
Hand	Right	n	13	5	12	
		%	92.8%	83.3%	100%	p=0.141
	Wrong	n	1	1	0	
		%	7.1%	16.6%	0%	
King	Right	n	6	5	10	
		%	42.8%	83.3%	83.4%	p=0.074#
	Wrong	n	8	1	2	
		%	57.1%	16.6%	16.6%	

* Statistically significant

# Trend towards a statistical significance

**Caption:** n = number of children; % = percentage; GI = Group 1; GII = Group 2; GIII = Group 3; p≤0.05

We associated the number of correct and incorrect answers in the IPRF with pictures with the participants’ age in each group, using Fisher's Exact test. The analysis revealed a significant difference – i.e., the dependence of the variables in seven of the 25 words, and a trend towards a significance in three of them if they are analyzed by many evaluators. The words with a significant difference in the association between age and test results were “ice”, “knife”, “cow”, “key”, “mouse”, “dog”, and “sun”; while the words with a trend to a significance were “ring”, “flower”, and “king”.

## DISCUSSION

DS is a genetic disorder characterized by the presence of an extra copy of chromosome 21 or the presence of an excess of genetic material. There is a predominance of males born with DS^(13)^, which agrees with the data collected in this study, as it had more male (54%) than female participants (46%). However, other authors report an equal number of boys and girls born with DS. Hence, sex seems to be a variable influenced by the type of research and the region where it is carried out^(14)^.

DS is known to cause global developmental delay. Children with this syndrome have some impairments that may hinder language and speech development. Most (95%) of this sample have speech-language-hearing follow-up, such as early speech and language stimulation and/or rehabilitation, to minimize their acquisition limitations and improve their development^(15,16)^.

The literature points out that 40% of people with DS have congenital heart disease; 100%, hypotonia; 50 to 70%, hearing problems; 15 to 20%, visual impairments; 1 to 10%, cervical spine problems; 15%, thyroid disorders; 5 to 10%, neurological problems; and, in an unknown percentage, obesity and premature aging^(14)^. We found 5% of comorbidities in the present study – one case of thyroid problems and another of physical disability in the upper limb.

Chronic otitis media and, consequently, conductive hearing loss are very common characteristics of this syndrome^(3,4,17)^. Even though 78% of this study participants’ parents/guardians reported no occurrence of otitis, 65% of the sample had an abnormal tympanic-ossicular chain. The most recurrent tympanometric curves were types B and C, followed by As and Ad in one of the ears. Most of the sample refused to put on the headphones for the contra- (58%) and ipsilateral (49%) reflexes. Nevertheless, 47.5% of them had normal speech recognition thresholds. Thus, the abnormal result in the immittance test suggests the onset or end of some middle ear or upper airway problem in these children, despite their normal thresholds.

Our findings do not suggest the presence of sensorineural hearing loss. This, however, cannot be confirmed because some participants refused to have the bone vibrator placed on them, while those who accepted it did not understand the task. The prevalence of sensorineural hearing loss in the first decade of life of children with DS is 4.4% to 8.1%, which increases from the second decade of life on because of the onset of early presbycusis and sequelae of persistent and evolving middle ear pathology^(14,18)^.

Only 32 of the 41 participants responded to the picture-based speech test. Therefore, we reduced the total sample n for its result analysis.

The picture-based speech perception test was applicable to normal-hearing children aged 2 years to 4 years and 11 months^(10)^ – which was also verified in this study regarding children with DS in the same age range (GI).

The test was applied to only six children in GII due to the difficulty recruiting DS individuals in this age group – the smallest one in this study. Despite being older than GI, they refused to undergo the assessment and repeated many words. Which on the other hand did not occur in GIII, as all these children were able to answer the test without difficulty.

In this study, the number of correct answers in the picture-based speech perception test increased with the child's age – which was also observed in a study that applied the same test to hearing-impaired children in the same age range^(19)^. Studies have shown that, as children grow older, they get more correct answers in relation to auditory stimulation time while word recognition difficulties decrease. This takes place because they auditorily recognize the word and associate it with the picture^(20,21)^.

Children with DS were able to respond to the picture-based speech assessment, although they had a short attention span and lexicon (as reported by the participants' relatives) and tried to speak the words. Of the 14 children in GI, 57.15% did not know the word/picture “ice”, 7.14% did not know the words/pictures “mouse”, “grape”, “train”, and “sun”, and 21.42% did not know the word “king”, according to their parents/guardians. Errors occurred in all the 25 words used in the test, regardless of age group. This can be explained by their cognitive and language development delay and limited lexicon/vocabulary, unlike children with typical development^(20,21)^.

The words that showed a significant difference in the hit/error association with age group were “ice”, “knife”, “cow”, “key”, “rat”, “dog” and “sun”. The words “flower” (p=0.061), “king” (p=0.074), and “ring” (p=0.097) had a trend towards a significance in this study, as they neared the significance level (p=0.05). If there had been a larger n, these words would probably have a significant difference.

The picture-based speech perception test was used as an assessment tool in studies with normal-hearing and hearing-impaired individuals^(10,19)^. They observed, in both studies, that the word “dog” likewise had a statistically significant difference, while “ice”, “knife”, and “rat” did so in the study with hearing-impaired individuals^(19)^. Since the three studies targeted different populations, it can be assumed that children mistook different words because they are not at the same developmental stages and may have many facilitating variables – e.g., stimulation from the family and school, adequate health conditions, and so on. Vocabulary acquisition is greatly motivated by lexicon construction data, environmental stimuli, frequency of occurrence, and familiarity.

Of the 32 participants, 75% missed the word “ice” – which makes it the word with the most errors in the test, with a statistically significant difference when associated with the numbers of hit/errors per age group (p=0.042). In GI, 92.85% of the children missed this word; in GII, 83.33%, and in GIII, 50%. This finding reveals either this population’s small vocabulary^(20)^ or this word/picture’s infrequent use, making it unfamiliar to these children.

In all groups, we observed a tendency of children to point to their own body when the words “hand”, “foot”, “eye”, and “mouth” were spoken, and even point to their shoes when they heard “tennis”; point upwards when they heard “sun”, make gestures and onomatopoeia for “motorcycle”. To compensate for their delayed oral production, many children use gestural communication for longer to make themselves understood^(22)^. Children with DS have difficulties understanding the assessment instructions, which is explained by the expressive language and cognitive delay^(23)^. Therefore, this population needs the support of pictures in assessments.

Another word with statistically significant evidence is “knife” (p=0.05). The errors in this word are explained by the similar sound traces between “knife” and “cow” (in Portuguese, “*faca*” and “*vaca*”); moreover, both words belong to the same picture sheet and are said close one to the other. We observed that when the word “knife” was said, the children often pointed to the “cow” – this population tends to make such phoneme substitutions^(24)^.

The word “dog” had the third most errors (53%) because, for the vast majority of children, the animal in question is named either “woof-woof” or “dog”. The caregivers addressed this issue when we were conducting the test. Likewise, errors regarding this word were frequent and significant both in the study that developed the test and in the one with hearing-impaired children^(10,19)^. Hence, the errors may not result from not knowing the animal in question or not having heard it, but rather from not knowing the specific word used in the test.

We compared this study participants’ performance with that of children with normal hearing and typical neurodevelopment^(10)^ and noticed that the highest percentage of correct answers, in both groups, were for the words “hand”, “house”, and “frog”. This can be explained by the groups’ chronological age in both studies – the normal-hearing children were younger –, as the receptive and expressive lexicon acquisition is delayed and below the expected for the chronological age in children with DS^(15,25)^.

## CONCLUSION

The picture-based speech perception test efficiently assessed children with DS. Further benefits include its understandability by this population and objective and clear result interpretation. Thus, it helps systematize the follow-up of children with phonological deviations or nonverbal children.
