# Supplementary material for: Figure-based speech perception test: applicability in children with Down syndrome
Source: Codas. 2022 Jan 7;34(3):e20200204. doi: 10.1590/2317-1782/20212020204 (PMC9769426; doi:10.1590/2317-1782/20212020204)
Supplement: Translation

# Translation: Teste de percepção de fala com figuras: aplicabilidade em crianças com síndrome de Down

**Authors:** Beatriz Nascimento Gonçalves, Isabela Raymundini Lorenssete, Nicolle Oliveira Tomé, Ana Lúcia Rios Mota, Cristiane Fregonesi Dutra Garcia, Ana Cláudia Mirândola Barbosa Reis

**Affiliations:** 1 Curso de Fonoaudiologia, Departamento de Fonoaudiologia, Faculdade de Medicina – FM, Universidade Federal do Rio de Janeiro – UFRJ - Rio de Janeiro (RJ), Brasil.; 2 Curso de Fonoaudiologia, Departamento de Ciências da Saúde, Faculdade de Medicina de Ribeirão Preto – FMRP, Universidade de São Paulo – USP - Ribeirão Preto (SP), Brasil.

**Keywords:** Percepção auditiva, Criança, Audição, Síndrome de Down, Fonoaudiologia, Percepção da Fala

## Abstract

**Objetivo:**

Verificar a aplicabilidade do teste de percepção de fala com figuras em crianças com síndrome de Down.

**Método:**

Estudo observacional, descritivo e prospectivo, realizado em dois centros fonoaudiológicos, aprovado pelos Comitês de Ética em Pesquisa sob número 82522217.5.0000.5440 e 79510317.8.0000.5257. Participaram 41 crianças com síndrome de Down, de ambos os sexos, com idade entre dois anos e dez anos e 11 meses, as quais foram divididas em três grupos: GI (dois a quatro anos e 11 meses); GII (cinco a sete anos e 11 meses) e GIII (oito a dez anos e 11 meses). Foram realizados os procedimentos de anamnese, meatoscopia, audiometria tonal liminar, teste de limiar de recepção de fala com figuras e imitanciometria. Para a análise estatística, Teste Exato de Fisher com nível de significância de 5%.

**Resultados:**

Ao ser analisado o número de acertos e erros, em relação à idade cronológica, foi encontrada significância para sete palavras: gelo, faca, vaca, chave, rato, cão e sol. Foi analisado, posteriormente, o desempenho no teste de fala com figuras, dos participantes desse estudo e o desempenho dos participantes do estudo que elaborou e validou este teste. Foi observado que, quando se equiparou a porcentagem de acertos nos dois grupos, as palavras com maior ocorrência de acertos foram: mão, casa e sapo.

**Conclusão:**

O teste aplicado nesse estudo proporciona a interpretação do resultado de forma clara e objetiva e independe da produção verbal da criança.

## INTRODUÇÃO

A síndrome de Down (SD) é uma alteração genética, caracterizada pela presença de uma cópia extra do cromossomo 21 ou excesso do material genético deste cromossomo^(1)^. Estudos demonstram que essa síndrome ocorre em um para cada 1000 nascidos vivos^(2)^. As características clínicas mais encontradas são: comprometimento intelectual, hipotonia muscular, fissura palpebral oblíqua, aumento da vascularização, microcefalia e occipital achatado. O acúmulo de muco pode resultar em infecções das vias aéreas superiores e consequente aumento dos quadros de otites médias^(3)^. Outro fator que pode contribuir para o aumento das otites é a disfunção ou comprometimento de orelha média, frequente nessa população. Este fator está relacionado com as malformações anatômicas, tais como tuba auditiva anormal, tecido mesenquimal persistente na cavidade timpânica, estenose do canal auditivo externo e hipoplasia da mastoide^(4)^.

Os primeiros anos de vida são importantes para o desenvolvimento da linguagem e crianças com otite média recorrente podem apresentar alterações nesse desenvolvimento, com prejuízos na aprendizagem escolar^(5)^. A audição é de suma importância para aquisição da linguagem oral. Como pré-requisitos para a aquisição normal da linguagem, tem-se a integridade anatômica e funcional do sistema auditivo periférico e central e as exposições às experiências auditivas. Os primeiros anos de vida, principalmente os seis primeiros meses, são considerados o período crítico para o desenvolvimento das habilidades auditivas. Em crianças ouvintes, o desenvolvimento e maturação auditiva seguem uma sequência padronizada de comportamentos que evoluem desde o nascimento até os dois anos de idade, que são as habilidades de detecção, discriminação, localização, reconhecimento e compreensão auditiva^(6,7)^.

Crianças com SD podem ter comprometimento intelectual, que somado ao prejuízo auditivo, podem levar a dificuldades no desenvolvimento da linguagem e fala. As crianças precisam da via auditiva integra para ter acesso a uma informação clara e, consequentemente, dar significado ao que escutam. A privação sensorial impede que o desenvolvimento das habilidades auditivas aconteça em seu curso natural^(8)^. Para essa população, o comprometimento auditivo ao longo da infância pode acarretar em problemas no desenvolvimento das habilidades auditivas e, consequentemente, processamento auditivo alterado^(9)^.

Avaliar as estruturas auditivas e a sua funcionalidade é um desafio na infância, principalmente na criança com SD. Há vários procedimentos e técnicas utilizados na investigação, desde os comportamentais aos objetivos. Com vistas a tornar a avaliação de fala mais lúdica e eficaz, foi desenvolvido um instrumento gráfico para a realização do teste de percepção de fala com crianças, de rápida e fácil aplicação, que demonstrou eficiência em atender aos objetivos na população infantil, pois possibilita a aplicação do teste independente da capacidade de verbalização^(10)^.

Os testes de percepção da fala, designados para crianças, devem usar palavras familiares e sua apresentação por meio de viva voz é mais adequada, por facilitar a apresentação dos estímulos durante o período de sua atenção. Faz-se necessária a elaboração de protocolos e procedimentos padronizados que avaliem aspectos específicos da percepção dos sons da fala em português^(11)^. A criança com SD pode apresentar um desenvolvimento de linguagem e fala deficitário, prejudicado por comprometimento cognitivo e/ou por uma alteração auditiva. Instrumentos de avaliação lúdicos, de fácil manuseio e que favoreça respostas quantitativa/qualitativa devem ser valorizados. Por isso, o presente estudo se propôs a verificar a aplicabilidade do teste de percepção de fala com figuras, em crianças com SD.

## MÉTODO

A pesquisa foi aprovada pelos Comitês de Ética em Pesquisa (CEP) das instituições proponentes do estudo, sob números CAAE 82522217.5.0000.5440 e 79510317.8.0000.5257.

O desenho do estudo é do tipo observacional, descritivo, prospectivo. Todos os participantes e seus responsáveis assinaram o Termo de Assentimento e de Consentimento Livre e Esclarecido;

Foram avaliadas crianças com diagnóstico de síndrome de Down, de ambos os sexos, na faixa etária entre dois anos e dez anos de idade. As crianças foram divididas em três grupos pelo critério de idade. O grupo GI foi composto por crianças de dois anos a quatro anos e 11 meses, o GII por crianças de cinco anos a sete anos e 11 meses e o GIII por crianças de oito anos a 10 anos e 11 meses.

As crianças que não se enquadraram nos critérios de inclusão e aquelas cujos responsáveis não concordaram em participar ou se recusaram em terminar as avaliações, foram excluídas.

O estudo consistiu-se de diversas etapas, sendo a primeira, uma anamnese audiológica com os pais/responsáveis, a fim de se obter dados da história médica e otológica da criança. Em seguida, com o auxílio de um otoscópio, uma inspeção do meato acústico externo e da membrana timpânica foi realizada, para a confirmação de viabilidade de execução da avaliação audiológica. Após, a audiometria tonal liminar lúdica, com todos os participantes, foi realizada em cabina acusticamente tratada, nas frequências de 500, 1000, 2000 e 4000 Hertz (Hz). Com o uso do audiômetro, o estímulo *warble* foi apresentado de forma descendente, em degraus de 10 dB, e ascendente, em degraus de 5 dB na pesquisa dos limiares auditivos. Consideramos o limiar audiométrico dentro dos padrões de normalidade, segundo Organização Mundial da Saúde^(12)^ e não nível de resposta mínima. Os procedimentos foram realizados em sistema de campo livre a 0º azimute e a caixa posicionada a 60 centímetros (cm) do pavilhão auricular, quando os participantes não aceitaram a colocação dos fones supra-aurais. Com objetivo de confirmar os limiares tonais, foi realizado o Limiar de Reconhecimento de Fala (LRF), por meio de ordens simples, como por exemplo: “Cadê a cabeça”, “Manda beijo”, “Dá tchau”, em intensidade inicial de 40 dBNS (deciBel Nível de Sensação) da média tritonal identificada na avaliação audiológica, pelo método descendente/ascendente. Posteriormente, com a mesma intensidade, foi aplicado o Índice Percentual de Reconhecimento de Fala (IPRF) com figuras^(10)^, que constitui-se em cinco pranchas teste e uma prancha treino, com seis figuras cada uma, correspondentes a palavras monossílabas e dissílabas, na qual as crianças deveriam apontar a figura dita pelo examinador. Em cada prancha eram ditas apenas cinco palavras, a fim de que a última não fosse selecionada por exclusão. Para este procedimento, um segundo examinador ou o próprio responsável pela criança, devidamente orientado, dentro da cabina, realizou a troca das pranchas, de acordo com a apresentação das palavras. Um treino prévio com uma lista de cinco palavras (prancha treino) foi realizado. A lista de palavras foi aplicada, sem pausas entre os fonemas, com repetição de apenas uma vez, se necessário. Foram utilizadas frases introdutórias para a apresentação da lista de figuras, como por exemplo: Mostre a(o) _______, e Cadê a(o) _______. Foi utilizado anteparo, para impedir o apoio de leitura orofacial, como no teste de IPRF convencional. Após a avaliação audiológica, a criança foi conduzida para a realização da imitanciometria, na qual se verifica o funcionamento do sistema tímpano-ossicular e se realiza pesquisa dos reflexos acústicos, contra e ipsilaterais. Para a análise estatística, foram utilizados o *software R Studio* e o Teste Exato de Fisher com nível de significância de 5%.

## RESULTADOS

A amostra da pesquisa foi composta por 41 crianças com síndrome de Down, com idade entre dois anos e dez anos e 11 meses. Do total da amostra, 25 crianças foram avaliadas no Setor de Fonoaudiologia no Hospital Universitário Clementino Fraga Filho (HUCFF) da Universidade Federal do Rio de Janeiro (UFRJ) e 16 crianças no Centro Especializado de Otorrinolaringologia e Fonoaudiologia do Hospital das Clínicas da Faculdade de Medicina de Ribeirão Preto da Universidade de São Paulo (CEOF – HCFMRP – USP). As crianças foram divididas em três grupos, segundo idade cronológica. O grupo GI foi composto por crianças de dois anos a quatro anos e 11 meses, o GII por crianças de cinco anos a sete anos e 11 meses e o GIII, por crianças de oito anos a dez anos e 11 meses. O grupo com o maior número de crianças participantes foi o GI com 21 crianças (51%), seguido pelo GIII, composto por 12 crianças (29%) e, por último, o GII com 8 crianças (20%). Na Tabela 1 estão os dados descritivos (porcentagem, média, mediana, desvio padrão e valores mínimos/máximos) em relação à idade das crianças do estudo, por grupo.

**Tabela 1:** Dados descritivos em relação à idade dos participantes, por grupo, em anos (n=41)

Grupos	n	(%)	Média (anos)	DP (anos)	Mínimo (anos)	Mediana (anos)	Máximo (anos)
GI	21	51%	3,2	0,88	2	3	4,11
GII	8	20%	6,3	0,75	5,1	6,5	7,4
GIII	12	29%	9,4	0,82	8	9,2	10,6

**Legenda: n** = número de crianças; (%) = porcentagem; DP = desvio padrão; GI = Grupo 1; GII = Grupo 2; GIII = Grupo 3

Os grupos foram compostos por crianças de ambos os sexos. Apenas GIII foi constituído mais por meninas (37%). No total, participaram 22 meninos (54%) e 19 meninas (46%).

Não foi possível realizar audiometria tonal na maioria dos participantes (60%), pois muitos não entendiam como deveriam realizar o procedimento, talvez pelo atraso de desenvolvimento cognitivo e de linguagem que podem apresentar; porém, em 73% (n=30), foi possível realizar o Limiar de Reconhecimento de Fala (LRF) e desses, a maioria (51%) estava com o limiar dentro da normalidade. Os dados da avaliação audiológica podem ser observados na Tabela 2.

**Tabela 2:** Dados da avaliação audiológica realizada pré-aplicação do teste de fala com figuras

Variáveis		n=41	(%)
Audiometria em Campo Livre	15 dB	16	40%
Limiares auditivos OD	Não realizado	15	36%
	Até 15 dB	5	12%
	Maior que 15 dB	5	12%
Limiares auditivos OE	Não realizado	15	36%
	Até 15 dB	2	5%
	Maior que15 dB	8	19%
LRF OD	Não realizado	11	27%
	Até 15 dB	21	51%
	Maior que15 dB	9	22%
LRF OE	Não realizado	11	27%
	Até 15 dB	21	51%
	Maior que15 dB	9	22%

**Legenda:** n = número de crianças; (%) = porcentagem; OD = orelha direita; OE = orelha esquerda; LRF = Limiar de Reconhecimento de Fala

Em relação aos resultados da timpanometria, cerca de 65% da amostra apresentou alteração no sistema tímpano-ossicular. As curvas timpanométricas de maior ocorrência foram do tipo “B” e “C”, seguidas pelas “Ar” e “Ad”, em uma das orelhas. Em 20% dos participantes, foram identificadas condições de normalidade de orelha média (curva tipo “A”) e 15% da amostra, não permitiu a realização do exame. Muitos da amostra não permitiram a colocação do fone para a pesquisa dos reflexos acústicos contralaterais (58%) e ipsilaterais (49%). Na Tabela 3 estão representados os dados referentes à avaliação de orelha média.

**Tabela 3:** Dados da avaliação timpanométrica e pesquisa de reflexos da amostra estudada

Variáveis		n=41	(%)
Timpanometria OD	Curva tipo A	8	20%
	Curva tipo B	14	34%
	Curva tipo C	9	21%
	Curva tipo Ar	3	7%
	Curva tipo Ad	1	3%
	Não realizada	6	15%
Timpanometria OE	Curva tipo A	9	21%
	Curva tipo B	13	32%
	Curva tipo C	8	20%
	Curva tipo Ar	3	7%
	Curva tipo Ad	1	3%
	Não realizada	7	17%
Reflexos ipsilaterais OD	Não realizado	20	49%
	Presente	6	15%
	Ausente	15	36%
Reflexos ipsilaterais OE	Não realizado	20	49%
	Presente	5	12%
	Ausente	16	39%
Reflexos contralaterais OD	Não realizado	24	58%
	Presente	6	15%
	Ausente	11	27%
Reflexos contralaterais OE	Não realizado	24	58%
	Presente	6	15%
	Ausente	11	27%

**Legenda:** n = número de crianças; (%) = porcentagem; OD = orelha direita; OE = orelha esquerda

Dos 41 participantes do estudo, apenas 32 responderam ao teste de fala com figuras; sendo assim, o **n** total da amostra para a análise do resultado foi reduzido. Na Tabela 4, estão descritas (média, mediana, desvio padrão, valores mínimos e máximos) das porcentagens de acertos obtidos no IPRF com figuras, dividido por grupo. Quando se analisou o desempenho individual dos grupos, foi observado um maior índice de acertos no grupo GIII (90%), seguido pelo GII e GI. Quando se analisou o desempenho individual dos grupos, foi observado um maior índice de acertos no grupo GIII (90%), seguido pelo GII e GI.

**Tabela 4:** Descrição dos acertos e erros, por palavra, do IPRF com figuras da amostra (n=32)

Figuras		Acerto	Erro
	n	28	4
Pato	%	87,50%	12,50%
	n	27	5
Bola	%	84,40%	15,60%
	n	27	5
Tênis	%	84,40%	15,60%
	n	26	6
Dedo	%	81,20%	18,70%
	n	29	3
Casa	%	90,60%	9,40%
	n	27	5
Gato	%	84,40%	15,60%
	n	8	24
Gelo	%	25%	75%
	n	14	18
Faca	%	43,80%	56,20%
	n	25	7
Vaca	%	78,10%	21,90%
	n	29	3
Sapo	%	90,60%	9,40%
	n	18	14
Zebra	%	56.2%	43,80%
	n	23	9
Chave	%	71,80%	28,20%
	n	24	8
Moto	%	75%	25%
	n	27	5
Leão	%	84,40%	15,60%
	n	21	11
Rato	%	65,60%	34,40%
	n	17	15
Anel	%	53,10%	46,90%
	n	30	2
Olho	%	93,70%	6,30%
	n	23	9
Uva	%	71,80%	28,20%
	n	27	5
Pé	%	84,40%	15,60%
	n	23	9
Trem	%	71,80%	28,20%
	n	15	17
Cão	%	46,90%	53,10%
	n	26	6
Flor	%	81,20%	18,70%
	n	22	10
Sol	%	68,70%	31,30%
	n	30	2
Mão	%	93,70%	6,30%
	n	21	1
Rei	%	65,60%	34,40%

**Legenda:** (n) = número; (%) = porcentagem

Em relação ao desempenho no teste em estudo, por palavra, da amostra constituída por 32 participantes, não observou-se 100% de acerto. Na Tabela 5, estão os dados descritivos em relação ao desempenho no IPRF com figuras, número e porcentagem de acertos e erros, valores de frequência e percentual, por palavra. Pode-se bservar que nas palavras: olho, mão, casa, sapo, pato, as crianças tiveram melhor desempenho; diferente do que aconteceu nas palavras gelo, faca, cão, anel e zebra, (na ordem de maior para a menor ocorrência), nas quais as crianças tiveram maiores dificuldades em reconhecer a figura e o índice de erros foi, consequentemente, maior.

**Tabela 5:** Associação do número de acertos e erros no IPRF com figuras, com a idade dos participantes do GI, GII e GIII (n=32)

Figuras						Teste Exato de Fisher
				Grupos		
			GI	GII	GIII	
			(n=14)	(n=6)	(n=12)	
Pato	Acerto	n	12	5	11	
		%	85,70%	83,30%	91,70%	p=1,000
	Erro	n	2	1	1	
		%	14,20%	16,60%	8,30%	
Bola	Acerto	n	10	5	12	
		%	71,40%	83,30%	100%	p=0,397
	Erro	n	2	1	0	
		%	14,20%	16,60%	0%	
Tênis	Acerto	n	10	5	12	
		%	71,40%	83,30%	100%	p=0,136
	Erro	n	4	1	0	
		%	28,50%	16,60%	0%	
Dedo	Acerto	n	9	6	11	
		%	64,20%	100%	91,70%	p=0,149
	Erro	n	5	0	1	
		%	35,70%	0%	8,30%	
Casa	Acerto	n	12	5	12	
		%	85,70%	83,30%	100%	p=0,390
	Erro	n	2	1	0	
		%	14,20%	16,60%	0%	
Gato	Acerto	n	10	5	12	
		%	71,40%	83,30%	100%	p=0,136
	Erro	n	4	1	0	
		%	28,50%	16,60%	0%	
Gelo	Acerto	n	1	1	6	
		%	7,10%	16,60%	50%	p=0,042*
	Erro	n	13	5	6	
		%	92,80%	83,30%	50%	
Faca	Acerto	n	2	3	9	
		%	14,20%	50%	75%	p=0,005*
	Erro	n	12	3	3	
		%	85,70%	50%	25%	
Vaca	Acerto	n	9	4	12	
		%	64,20%	66,70%	100%	p=0,045*
	Erro	n	5	2	0	
		%	35,70%	33,40%	0%	
Sapo	Acerto	n	12	5	12	
		%	85,70%	83,30%	100%	p=0,390
	Erro	n	2	1	0	
		%	14,20%	16,60%	0%	
Zebra	Acerto	n	5	4	9	
		%	35,70%	66,70%	75%	p=0,146
	Erro	n	9	2	3	
		%	64,20%	33,40%	25%	
Chave	Acerto	n	8	3	12	
		%	57,10%	50%	100%	p=0,012*
	Erro	n	6	3	0	
		%	42,80%	50%	0%	
Moto	Acerto	n	9	5	10	
		%	64,20%	83,30%	83,40%	p=0,563
	Erro	n	5	1	2	
		%	35,70%	16,60%	16,60%	
Leão	Acerto	n	10	5	12	
		%	71,40%	83,30%	100%	p=0,136
	Erro	n	4	1	0	
		%	28,50%	16,60%	0%	
Rato	Acerto	n	5	4	12	
		%	35,71%	66,70%	100%	p=0,001*
	Erro	n	9	2	0	
		%	64,20%	33,40%	0%	
Anel	Acerto	n	5	3	9	
		%	35,70%	50%	75%	p=0,097^#^
	Erro	n	9	3	3	
		%	64,20%	50%	25%	
Olho	Acerto	n	12	6	12	
		%	85,70%	100%	100%	p=1,000
	Erro	n	2	0	0	
		%	14,20%	0%	0%	
Uva	Acerto	n	8	5	10	
		%	57,10%	83,30%	83,40%	p=0,349
	Erro	n	6	1	2	
		%	42,80%	16,60%	16,60%	
Pé	Acerto	n	11	5	11	
		%	78,20%	83,30%	91,70%	p=0,229
	Erro	n	3	1	0	
		%	21,40%	16,60%	0%	
Trem	Acerto	n	8	5	10	
		%	57,10%	83,30%	83,40%	p=0,349
	Erro	n	6	1	2	
		%	42,80%	16,60%	16,60%	
Cão	Acerto	n	5	1	9	
		%	35,70%	16,60%	75%	p=0,042*
	Erro	n	9	5	3	
		%	64,20%	83,30%	25%	
Flor	Acerto	n	9	5	12	
		%	64,20%	83,30%	100%	p=0,061^#^
	Erro	n	5	1	0	
		%	35,70%	16,60%	0%	
Sol	Acerto	n	6	5	11	
		%	42,80%	83,30%	91,70%	p=0,022*
	Erro	n	8	1	1	
		%	57,10%	16,60%	8,30%	
Mão	Acerto	n	13	5	12	
		%	92,80%	83,30%	100%	p=0,141
	Erro	n	1	1	0	
		%	7,10%	16,60%	0%	
Rei	Acerto	n	6	5	10	
		%	42,80%	83,30%	83,40%	p=0,074#
	Erro	n	8	1	2	
		%	57,10%	16,60%	16,60%	

* estatisticamente significante

# tendência a ser estatisticamente significante

**Legenda:** n = número de crianças; % = porcentagem; GI = Grupo 1; GII = Grupo 2; GIII = Grupo 3; p≤0,05

A análise segundo o Teste Exato de Fisher para associar o número de acertos e erros no IPRF com figuras com a idade dos participantes de cada grupo, revelou diferença significativa, ou seja, o teste evidencia a dependência das variáveis para sete, das 25 palavras e três com tendência a ser significativa, caso seja analisada sob um número maior de avaliados. As palavras que evidenciaram uma diferença significativa na associação entre idade e resultado do teste foram: gelo, faca, vaca, chave, rato, cão e sol; enquanto as palavras com uma tendência a serem significativas foram: anel, flor e rei.

## DISCUSSÃO

A síndrome de Down é uma alteração genética caracterizada pela presença de uma cópia extra do cromossomo 21 ou presença de excesso de material genético. Há uma dominância do nascimento de crianças do sexo masculino na (SD)^(13)^, o que está de acordo com os dados levantados neste estudo, pois o número de participantes do sexo masculino (54%) foi maior que o sexo feminino (46%). Entretanto, outros autores relatam que há igualdade entre o nascimento de meninos e meninas com SD, sendo que a variável sexo parece ser influenciada pelo tipo da pesquisa e região onde a mesma é realizada^(14)^.

Sabe-se que a síndrome de Down leva a um atraso no desenvolvimento global. Crianças com essa síndrome apresentam alguns comprometimentos que podem levar a dificuldades no desenvolvimento da linguagem e fala. A maioria (95%) dessa amostra faz algum acompanhamento fonoaudiológico, como estimulação precoce de fala e linguagem e/ou reabilitação, a fim de minimizar as limitações em suas aquisições e melhorar o desenvolvimento destes processos^(15,16)^.

A literatura aponta que 40% dos portadores da síndrome apresentam cardiopatia congênita; 100%, hipotonia; 50 a 70%, problemas de audição; 15 a 20%, alterações visuais; 1 a 10%, alterações na coluna cervical; 15%, distúrbios da tireoide; 5 a 10%, problemas neurológicos e, em um percentual não estabelecido, aparecem os casos de obesidade e envelhecimento precoce^(14)^. Nesse estudo, verificou-se 5% de comorbidades, sendo um caso de alteração na tireoide e outro de deficiência física em membro superior.

Dentro das várias características dessa síndrome, a presença de otites médias crônicas e por consequência, perdas auditivas do tipo condutivas é muito comum nessa população^(3,4,17)^. Apesar de 78% dos responsáveis pelos participantes do estudo referirem não ocorrência de otites, 65% da amostra apresentou sistema tímpano-ossicular alterado. As curvas timpanométricas que mais apareceram foram do tipo “B” e “C”, seguidas pelas “Ar” e “Ad” em uma das orelhas. A maioria da amostra não permitiu a colocação do fone para a pesquisa dos reflexos contralaterais (58%) e ipsilaterais (49%). Entretanto, desses sujeitos, 47,5% estavam com o limiar de reconhecimento de fala dentro dos padrões da normalidade. Assim, a alteração na imitanciometria pode sugerir que estas crianças, apesar dos limiares estarem dentro da normalidade, podem estar com quadros de início ou fim de alguma alteração de orelha média ou de vias aéreas superiores.

Os achados deste estudo não sugeriram a presença de perda auditiva sensorioneural, mas também esse dado não pode ser confirmado, pois os participantes da pesquisa não aceitaram a colocação do vibrador ósseo e os que aceitaram não compreendiam a tarefa a ser realizada. Há uma prevalência de perda auditiva sensorioneural em crianças com SD, na primeira década de vida, de 4,4% a 8,1%, sendo que esta prevalência aumenta, a partir da segunda década de vida, em razão do aparecimento de uma presbiacusia precoce e de sequelas relacionadas à patologia da orelha média persistente e evolutiva^(14,18)^.

Dos 41 participantes do estudo, apenas 32 crianças responderam ao teste de fala com figuras, sendo assim o **n** total da amostra para a análise do resultado para esse teste foi reduzido.

A aplicabilidade do teste de percepção de fala com figuras foi possível em crianças normo-ouvintes na faixa etária de dois anos a quatro anos e 11 meses^(10)^, o que também foi verificado nesse estudo, com as crianças portadoras de SD de faixa etária equiparada (GI).

Em relação às crianças do GII, grupo de menor **n** desse estudo, o teste foi aplicado em apenas seis crianças, pela dificuldade de recrutar pessoas com SD nessa faixa etária. Apesar de ter idade maior que o GI, nesse grupo foram encontrados comportamentos de recusa à realização da avaliação e solicitação de muitas repetições das palavras. O mesmo não ocorreu no GIII, grupo no qual todas as crianças conseguiram responder ao teste, sem dificuldades.

Nesse estudo, o número de acertos no teste de percepção de fala com figuras teve um aumento, conforme a progressão da idade da criança; o que também foi observado num estudo em que o mesmo teste foi aplicado em crianças deficientes auditivas, na mesma faixa etária^(19)^. Estudos demonstraram um maior número de acertos em relação ao tempo de estimulação auditiva e menor dificuldade de reconhecimento de palavras, com o aumento da faixa etária das crianças, já que essas reconheceram auditivamente a palavra e associaram-na com a figura^(20,21)^.

As crianças com SD conseguiram responder à avaliação de fala com figuras, porém apresentaram tempo de atenção reduzido, tentativa de emissão oral das palavras e um léxico reduzido, segundo descrito pelos próprios familiares dos participantes. Das 14 crianças com SD (GI), 57,15% não conheciam a palavra/figura “gelo”, 7,14% não conheciam as palavras/figura “rato”, “uva”, “trem” e “sol” e 21,42% não conheciam a palavra “rei”, segundo seus responsáveis. Das 25 palavras utilizadas no teste, foi possível observar erros em todas as apresentadas, independente da faixa etária. Esses dados podem ser justificados pelo modo lento e atrasado que ocorre o desenvolvimento cognitivo e o de linguagem, assim como pela não expansão do léxico/vocabulário dessa população, como ocorre nas crianças com desenvolvimento típico^(20,21)^.

As palavras que evidenciaram uma diferença significativa na associação acertos/erros com a faixa etária dos grupos foram: “gelo”, “faca”, “vaca”, “chave”, “rato”, “cão” e “sol“. As palavras “flor” (p=0,061), ”rei” (p=0,074) e “anel” (p=0,097), foram consideradas, neste estudo, com tendência à significância, devido ao valor próximo ao considerado (p=0,05). Caso o **n** utilizado fosse maior, provavelmente estas palavras também se encaixariam com diferença significativa.

O teste de percepção de fala com figuras foi utilizado como instrumento de avaliação em estudo com normo-ouvintes e deficientes auditivos^(10,19)^. Observaram, em ambos os estudos, que a palavra “cão” também apareceu com diferença estatística significativa; as palavras “gelo”, “faca” e “rato” também apareceram com esta diferença, no estudo com deficientes auditivos^(19)^. Ao levar em conta que os três estudos tiveram como alvo, populações distintas, pode-se pensar que as crianças erraram palavras diferentes, pois, não estão em fases de desenvolvimento iguais e podem ter muitas variáveis facilitadoras, como estimulação do ambiente familiar e escolar, adequadas condições de saúde, dentre outras. Dados que favorecem a construção do léxico, sendo que os estímulos ambientais, a assiduidade e familiaridade de ocorrência são de suma importância para a aquisição de vocabulário.

Dos 32 participantes da pesquisa, 75% errou a palavra “gelo”, o que a torna a palavra com o maior número de erros do teste e com diferença estatisticamente significativa, ao ser associada aos números de acertos/erros por faixa etária (p=0,042); GI, 92,85% das crianças erraram esta palavra; GII, 83,33% e GIII, 50%. Este achado pode ser evidenciado pelo léxico reduzido dessa população e a alteração de vocabulário das mesmas^(20)^ ou a palavra/figura não é rotineiramente utilizada, assim como pouco familiar para essa população.

Em todos os grupos foi observado uma tendência das crianças a apontar partes do próprio corpo, quando as palavras “mão”, “pé”, “olho” e “boca” eram faladas e, até mesmo apontar para o sapato, na palavra “tênis”; apontar para cima, na palavra “sol”, realizar sinais e onomatopeias para a palavra “moto”. Para compensar o atraso de sua produção oral, muitas crianças fazem uso de comunicação gestual por mais tempo, com o objetivo de melhorar a compreensão pelo interlocutor^(22)^. Crianças com SD apresentam dificuldade no entendimento das ordens para a realização da avaliação, justificada pelo atraso cognitivo e de linguagem expressiva^(23)^; sendo assim, o apoio de figuras em avaliações para essa população é necessário.

Dentre as outras palavras que apresentaram uma evidência estatisticamente significativa, está a palavra “faca” (p=0,05). Os erros desta palavra se justificam, pois, os traços de sonoridade das palavras “faca” e “vaca” são muito parecidos e as duas palavras são ditas durante a aplicação da mesma prancha de figuras do teste realizado. Observamos, por inúmeras vezes, que ao ser dita a palavra “faca”, as crianças se dirigiam à imagem da “vaca”, o que é uma propensão dessa população, fazer trocas fonológicas^(24)^.

A palavra “cão” foi a terceira com o maior número de erros (53%). Isto se deve ao fato de que para a grande maioria das crianças, o animal em questão é intitulado como “auau” ou “cachorro”. Esta foi uma questão abordada pelos responsáveis ao realizarmos o teste. Os erros nesta palavra também foram frequentes e considerados significativos no estudo da elaboração do teste e também quanto aplicado em crianças deficientes auditivas^(10,19)^; ou seja, talvez os erros não sejam pelo não conhecimento do animal em questão, ou por não terem escutado, e sim pelo desconhecimento da palavra utilizada para a elaboração do teste.

Quando comparamos o desempenho dos participantes deste estudo com a população de normo-ouvintes com neurodesenvolvimento típico^(10)^, observamos que a porcentagem de acertos, nos dois grupos, de maior ocorrência foram para as palavras “mão”, “casa” e “sapo”. Fato este pode ser justificado ao consideramos a idade cronológica dos grupos dos dois estudos, sendo os normo-ouvintes mais jovens, pois a aquisição do léxico receptivo e expressivo é atrasada e abaixo do esperado para a idade cronológica nas crianças com SD^(15,25)^.

## CONCLUSÃO

O teste de percepção de fala com figuras, para avaliar crianças com SD, foi vantajoso e pode ser benéfico; pois, além de ser de fácil entendimento para essa população, é um material que proporciona a interpretação do resultado, de forma objetiva e clara. E assim, possibilita o acompanhamento sistematizado de crianças que não apresentem oralidade ou que apresentem desvios fonológicos.
